# Formulation Development and Toxicity Assessment of Triacetin Mediated Nanoemulsions as Novel Delivery Systems for Rapamycin 

**Published:** 2015

**Authors:** Hamideh Sobhani, Parastoo Tarighi, Seyed Nasser Ostad, Alireza Shafaati, Nastaran Nafissi-Varcheh, Reza Aboofazeli

**Affiliations:** a *Department of Pharmaceutics, School of Pharmacy, Shahid Beheshti University of Medical Sciences, Tehran, Iran.*; b *Department of Medical Biotechnology, School of Advanced Technologies in Medicine, Tehran University of Medical Sciences, Tehran, Iran. *; c *Department of Pharmacology and Toxicology, Faculty of Pharmacy, Tehran University of Medical Sciences, Tehran, Iran. *; d *Department of Pharmaceutical Chemistry, School of Pharmacy, Shahid Beheshti University of Medical Sciences, Tehran, Iran. *; e *Department of Pharmaceutical Biotechnology, School of Pharmacy, Shahid Beheshti University of Medical Sciences, Tehran, Iran. *

**Keywords:** Rapamycin, Nanoemulsion, Phase behavior, MTT test, TEER, Permeability test, Cell toxicity

## Abstract

The aim of this investigation was to design and develop nanoemulsions (NEs) as novel delivery systems for rapamycin. Phase behavior of quaternary systems composed of Traicetin (as oil), various surfactants and co-surfactants and water at different surfactant/co-surfactant weight ratios was investigated by the construction of phase diagrams. Formulations were taken from the o/w NE region of the phase diagrams, depending upon the extent of NE domain. The spontaneous emulsification method was used to prepare various formulations containing 1 mg/mL of the drug. The NEs were characterized and subjected to stability tests at various temperatures over 9-12 months. Cumulative drug release from the selected formulations was determined for a period of 48 h using a dialysis sac. The assay of rapamycin was carried out using an HPLC technique. The effect of NEs on the viability of SKBR-3 cells was evaluated by MTT assay. The integrity of Caco-2 cell monolayers was measured by Transepithelial Electrical Resistance (TEER) and the transport of rapamycin-loaded NEs across Caco-2 cell monolayers was then assessed. The uptake of NEs by SKBR-3 cells was also investigated using florescence microscopy. Maximum drug release was observed in case of 4 formulations prepared with Tween 80 and Tween 20. MTT test results revealed different toxicity of NEs for SKBR-3 cell line and TEER demonstrated that formulations containing Tween 20 caused a more considerable decrease in cell integrity in comparison with those prepared with Tween 80. The results obtained from cellular uptake experiments were in consistent with those obtained from TEER and cytotoxicity experiments.

## Introduction

Rapamycin (RAP), known as sirolimus, is a naturally occurring water-insoluble macrolide antibiotic with immunosuppressive properties and has been extensively administered to patients receiving organ transplants to prevent organ rejection (1-3). RAP has also revealed an impressive antitumor activity and it is a potent inhibitor of the proliferation of T and B cells (4-11). RAP is a lipophilic drug (Log P of around 4.3) which is practically insoluble in water (2.6 mg/mL) with no ionizable functional groups in the pH range between 1 and 10. Following oral administration, RAP is absorbed rapidly but poorly in a dose-dependent manner with 15% bioavailability. Its oral bioavailability is mainly affected by the first pass metabolism and p-glycoprotein (P-gp) pump efflux (12), resulting in a large inter and intra-patient variability in the oral bioavailability. Therefore, formulation of RAP into either an intravenous or oral dosage form remains challenging due to its water insolubility and low bioavailability. RAP is commercially marketed as an oral solution; however, its regular administration even in low doses (1–2 mg/day) can produce side-effects in humans.

Various approaches have been investigated to formulate RAP in an attempt to enhance its bioavailability, anti-proliferative and immunosuppressive activities and reduce its side effects. These approaches include its association to polymeric carriers (13-18), liposomes (19), polymeric micelles (20), and preparation of solid dispersion formulations (3). Microemulsions (MEs) and nanoemuslions (NEs) are two kinds of lipid-based formulations with droplet size in the nanometric range and have been the focus of much research due to their great long term physical stability, simplicity of their formation process and their high potential in pharmaceutical applications. These self-emulsifying systems are considered as promising technologies for enhancing the bioavailability of the poorly water-soluble drugs. Although NEs and MEs are generally described in the literature indiscriminately, they are fundamentally different particularly from thermodynamic stability point of view (21, 22). The formation, properties and physicochemical fundamentals regarding these two systems are well reviewed by Anton and Vandamme (23). Various definitions have been proposed in the literature regarding NEs. Generally accepted, NEs are transparent (or translucent) dispersions of oil and water, stabilized by an interfacial film of combined surfactant and co-surfactant molecules, having a droplet size in the range of 50-200 nm (the upper limit depends upon the authors). NEs, also referred to as miniemulsions, (24), are non-equilibrium systems with ultralow interfacial tensions and unlike microemulsions (which are thermodynamically stable), they possess a relatively high kinetic stability, with no apparent droplet flocculation and coalescence (21, 25, 26), so that they are sometimes described as “Approaching Thermodynamic Stability” (26). The large interfacial areas associated with the presence of nanosized droplets would influence the transport properties of the drug, an important factor in sustained and targeted drug delivery (27). These systems have high solubilization capacity compared to simple micellar solutions and their long term physical stability offers advantages over unstable dispersions. Ease of preparation, increasing solubility and improving mucosal permeability of poorly water soluble compounds (28, 29), and ability to protect drugs from hydrolysis and enzymatic degradation in physiologic conditions (30-35) are other advantages considered for NEs. Three methods may be applied for the NE preparation, namely high energy emulsification (using high-pressure homogenizers and ultrasound generators), low energy emulsification (stepwise addition of oil/water to a water/oil surfactant mixture or mixing all the components in the final composition) and the principle of phase inversion temperature (22, 25, 26, 36). 

The present investigation was planned to develop and characterize Triacetin-mediated o/w NEs as delivery systems for RAP. It was hypothesized that by loading RAP in NEs, its bioavailability and anti-proliferative effect would increase. Triacetin was selected as the oil phase for the drug solubilization. NEs permeability across Caco-2 cell monolayer and their cytotoxicity on SKBR-3 cell line were also evaluated.

## Experimental

*Materials*

Tween 80, Tween 20, Triacetin, *iso*-propanol, polyethylene glycol 400 (PEG 400), propylene glycol (PG), methanol and dimethyl sulfoxide (DMSO) were purchased from Merck Chemical Co. (Germany). Labrasol (caprylocaproyl macrogol- 8- glycerides), Transcutol (diethylene glycol monoethyl ether) were gifted by Gattefosse Co. (France). Cremophor RH 40 was provided as a gift from Osvah Pharmaceutical Co. (Tehran, Iran). Penicillin-streptomycin (Pen strep) 100X and RPMI 1640 medium were provided from Biosera (England). Dulbecco᾽s Modified Eagle’s Medium (DMEM-high glucose), fetal bovine serum (FBS), trypsin-ethylenediamine tetraacetic acid (EDTA), 4-(2-hydroxyethyl)-1-piperazineethanesulfonic acid (HEPES) and Hank᾽s Balanced Salt Solution (HBSS) were prepared from PAA Laboratories GmbH (Austria). Trypan blue (0.4% w/v in PBS) and 3-(4,5-dimethylthiazol-2-yl)-2,5-diphenyl tetrazolium bromide (MTT) were provided from Sigma (USA) and DMEM F12 was obtained from Atocel (Austria). Purified water was collected from a Millipore Milli-Q Plus Water Purification System (Millipore, France). Rapamycin (RAP; sirolimus, batch number SIRI0012) and Rapamune® were purchased from Euticals Spa Co. (Italy), and Pfizer (Netherlands), respectively.

*Construction of pseudo-ternary phase diagrams*

Appropriate amounts of the oil (Triacetin), surfactants (including Cremphor RH 40, Labrasol, Tween 20 and Tween 80) and co-surfactants (namely *iso*-propanol, PEG 400, PG and Transcutol) were weighed into screw-capped vials and the samples were mixed at room temperature until a clear solution was obtained. Phase diagrams were constructed by titrating these samples with aliquots of triple distilled water and stirring for a sufficiently long time to attain equilibrium. The course of each titration was monitored both visually for determining the clarity and through cross-polaroids in order to determine the boundaries of any birefringent liquid crystalline phase. The phase behavior of the systems was mapped on triangle phase diagrams with the top apex representing a fixed surfactant/co-surfactant weight ratio (R_sm_ of 1;1, 1:2 or 2:1) and the other two apices representing Triacetin and water. All mixtures produced optically transparent or translucent, non-birefringent, isotropic solutions at low oil and low water domains were termed o/w and w/o NEs, respectively. 

*High Performance Liquid Chromatography (HPLC)*

The samples were analyzed by a previously described HPLC method (37). Chromatographic analysis was performed on Knauer HPLC system (Germany), consisting of a pump (Smartline 1000), UV detector (Smartline 2500) and software (Chromgate V3.1.7). A reversed phase C8 column (MZ, 15 × 4.6 mm, 5 μm particle size, MZ Analysentechnik GmbH, Germany) was used for the separation. A proper guard column was also applied. Mobile phase was a mixture of methanol:water (80:20 v/v) which was freshly prepared and degassed each day. Column temperature was set at 57 °C (Knauer, Germany). The injection of samples was performed on a Reodyne injector equipped with a 20 μL loop. UV detector was set at 277 nm. The method was validated for RAP assay according to the accepted guidelines with respect to specificity, linearity, precision (intra/inter-day RSD) and accuracy. 

*Determination of rapamycin solubility in Triacetin*

Briefly, excess amounts of RAP were added to 1 mL of Triacetin. The mixtures were stirred for 72 h at room temperature to reach the equilibrium. After achieving equilibrium and removing the excess amounts of the drug, samples were centrifuged at 14000 rpm for 15 min. The supernatant was taken and filtered through a 0.2 μm membrane filter. The solubility of RAP in Triacetin was then determined by the HPLC method. 

*Preparation of rapamycin-loaded nanoemulsions*

Pseudo-ternary phase diagrams were constructed to obtain the components and their concentration ranges which can result in the formation of NEs. After the o/w NE regions in the phase diagrams were identified, those systems that indicated a relatively extended o/w NE domain and that allowed to choose an oil content for completely solubilizing the drug, were selected. Following the preparation and characterization of the selected blank NEs, those systems with the particle size in the range of less than 100 nm and polydispersity index (PDI) of less than 0.5 which were transparent/translucent after 72 h storage at room temperature, were chosen for loading the drug. RAP-NEs were prepared by the spontaneous emulsification technique according to the following briefly described procedure. The oil phase was separately prepared by mixing the appropriate amounts of Triacetin, a surfactant and a co-surfactant. To the resultant mixture, a specific amount of RAP was then added with constant stirring using a mechanical stirrer until a clear solution was obtained. At the final stage, the required amount of water was added in a drop wise fashion with gentle agitation for at least 20 min in order to obtain a transparent/translucent NE. For convenience purposes, 1 mL of NEs was prepared with a minimum weight of surfactant/co-surfactant mixture (40% w/w for the required amount of the oil) and 1 mg of RAP.

*Particle size measurement*

The particle size and PDI of all RAP-loaded NEs were determined at room temperature by photon correlation spectroscopy, using a Malvern Zetasizer (Nano-ZS; Malvern Instruments, Worcestershire, UK)., equipped with a Nano ZS^®^ Software for data acquisition and analysis. The analysis was performed three times to determine the mean values.

*Determination of zeta potential*

The zeta potential of undiluted drug-loaded NEs was determined using a Malvern Zetasizer (Nano-ZS; Malvern Instruments, Worcestershire, UK). All analyses were done in triplicate. 

*Transmission electron microscopy*

A transmission electron microscope (Philips CM30, Netherlands) was used to observe the two-dimensional, relative size morphology of RAP-loaded NE particles. A drop of the sample was directly deposited on the holey film grid and observed after drying. Images were then taken with magnification of 11000.

*In-vitro drug release*

The release profile of RAP from developed NEs was evaluated using the dialysis bag method. Dialysis bags (MWCO 12 KD, Iran) were soaked in distilled water for 24 h and kept under refrigeration until use. One milliliter of a NE formulation (containing 1 mg of RAP) was packed in each dialysis bag, then sealed with clips at both ends and placed in the release medium (100 mL water containing 0.05% w/v of Tween 80). The assembly was stirred at a speed of 100 rpm at 37±1 °C and the aliquots of 2 mL were withdrawn from the dissolution medium at predetermined time intervals for 48 h (1, 2, 4, 8, 12, 24, 36 and 48 h). An equivalent volume of fresh medium was added to maintain the volume of the medium at 100 mL and ensure the sink condition. The samples were analyzed for the drug content using an HPLC method as described above. 

*Stability tests*

Developed RAP-loaded NEs were subjected to stability tests. The samples were stored at 4, 25 and 40 ºC and the stability was observed over a period of 9-12 months. The NEs were evaluated for particle size, PDI, drug content and monitored for any phase separation.

*Cell Culture*

Live SKBR-3 cells (National Cell Bank of Iran, Code: 207) was purchased from Pasteur Institute of Iran (passage number 8). Cells were cultured in 25 cm^2 ^plastic flasks (Nunc, Denmark) in an enriched medium containing 84% v/v Dulbecco᾽s Modified Eagle’s Medium (DMEM high glucose), 15% v/v of fetal bovine serum (FBS) and 1% v/v of penicillin- streptomycin (100 IU/mL) and incubated in a controlled humidified atmosphere consisting of 5% CO_2_ and 95% air at 37 °C. The cultured medium was changed every second day. The cells were trypsinized with trypsin- EDTA (1X) and resuspended in the culture medium and kept at 37 °C. Cell with passage number up to 20 were used for cytotoxicity studies.

Caco-2 cell line (passage number 40-45) was used for the permeability studies. Cell were grown at 37 °C in 75 cm^2^ plastic flask in a controlled humidified atmosphere of 5% CO_2_ and 95% air, using 15 mL of a medium containing 50% v/v RPMI 1640 supplemented with 34% v/v of DMEM F12, 15% v/v FBS and 1% v/v penicillin- streptomycin (100 IU/mL) which was changed every second day. The cells were trypsinized with trypsin- EDTA (1X), passaged and maintained for seven days, until the desired confluency of 80-90 was reached. Cells with a passage number up to 20 were used for cytotoxicity studies.

*Cytotoxicity assay*

Cytotoxicity was assessed using MTT to evaluate the viability of the cells (38). Cells at the density of 1× 10^4^ viable cells per well were seeded in 96-well microplate and incubated for 48 h to allow cell attachment. The cells were incubated with blank NEs, RAP-loaded NEs and RAP methanolic solution in an atmosphere of 5% CO_2_ at 37°C for 48 h. After washing with phosphate buffer saline (PBS), cells were incubated with 20 μL of 5 mg/mL MTT solution at 37 °C. Following 4 h incubation, 100 μL of DMSO was then added to each well while stirring vigorously, in order to dissolve formazon crystals. To measure the absorbance of each well, enzyme-linked immunosorbent assay reader (Anthous 2020; Anthos Labtec Instruments, Salzburg, Austria) was employed at the wavelength of 550 nanometer . Cell viability was finally calculated as a percentage of the control. 

*Measurement of Transepithelial Electrical Resistance (TEER)*

Semipermeable polycarbonate filter inserts (Nunc 12- well Transwell plates, pore size 0.4 μm, surface area 1.13 cm^2^) were used to seed the Caco-2 cells. Filter inserts were precoated with 100 μL of diluted rat tail collagen (type 1) solution in 0.1N acetic acid in the ratio of 1:9, in an attempt to enhance the cell adhesion. Coated filter inserts were dried over nigh in an incubator and the cells (at the density of 4×10^5^ cells/cm^2^) were then seeded on polycarbonate filters (39, 40). Transepithelial electrical resistance (TEER) of Caco-2 cell monolayer was measured 21 days after seeding the cells on filter inserts. The integrity of the Caco-2 cell monolayers on the filter inserts was evaluated using an EVOM 2 voltohmmeter equipped with “chopstick” electrodes (World Precision Instrument, Sarasota, USA). Culture medium at both apical (500 μL) and basolateral sides (1500 μL) was changed every other day. Mean TEER values across the Caco-2 cell monolayers were calculated based on the following equation: 

TEER= (*R*_monolayer_- *R*_blank_) × *A *(W cm^2^ )

where *R*_monolayer_ indicates the resistance of Caco-2 monolayer, *R*_blank_ is the resistance of inserts without Caco-2 cell monolayer and *A *is the available surface area of the filter inserts. TEER measurements were also performed during the permeation study at the specific time intervals (0, 1, 2, 3, 4 and 8 h) and 24 h after the permeation test in order to evaluate the effects of NEs on opening the tight junction barriers.

*Transport study*

To conduct the transport experiments, cell were first washed twice with PBS and incubated with HBSS (pH 7.4) supplemented with 25 mM HEPES for 30 min. Once the transition buffer was removed, RAP-loaded NEs and RAP methanolic solution were added to the apical side of the monolayer at a final concentration of 1 mg/mL. The basolateral side of the monolayer contained only 1.5 mL of the transition buffer. All transport analyses were conducted at 37 °C in the apical (A) to basolateral (B) direction of the monolayer. Samples were taken at the predetermined time intervals (0, 1, 2, 3 and 4 h) from the basolateral side of the monolayer and analyzed with the HPLC method described above. The apparent permeability coefficient (P_app_) was finally calculated using the following equation:

P_app_= *Q */ (*A *× *t *× *C*_0_)

where *Q *is the cumulative drug amount in the receptor compartment, *A *represents the filter surface area, *t *is the sampling time and *C*_0_ indicates the initial drug concentration in the apical compartment.

*Cellular uptake of nanoemulsions*

SKBR-3 cells were cultivated at a density of 40000 cells per well in a 12-well plate and incubated at 37 °C in the presence of NEs loaded with coumarin 6 and also methanolic solution of coumarin 6 as the control at the concentration of 0.5 μg/mL. After 6 h, cells were washed three times with PBS and the cellular uptake of coumarin 6-loaded NEs were then inspected and evaluated using fluorescence microscopy. The intensity of fluorescent color in each obtained image was finally determined.

Statistical analysis

Data are reported as mean ± SD. Statistical analysis of differences between the samples was performed using one-way ANOVA and an appropriate post-test, if necessary. A 0.05 level of probability was taken as the level of significance.

## Results and Discussion

*Phase behavior studies*

**Figure 1:**
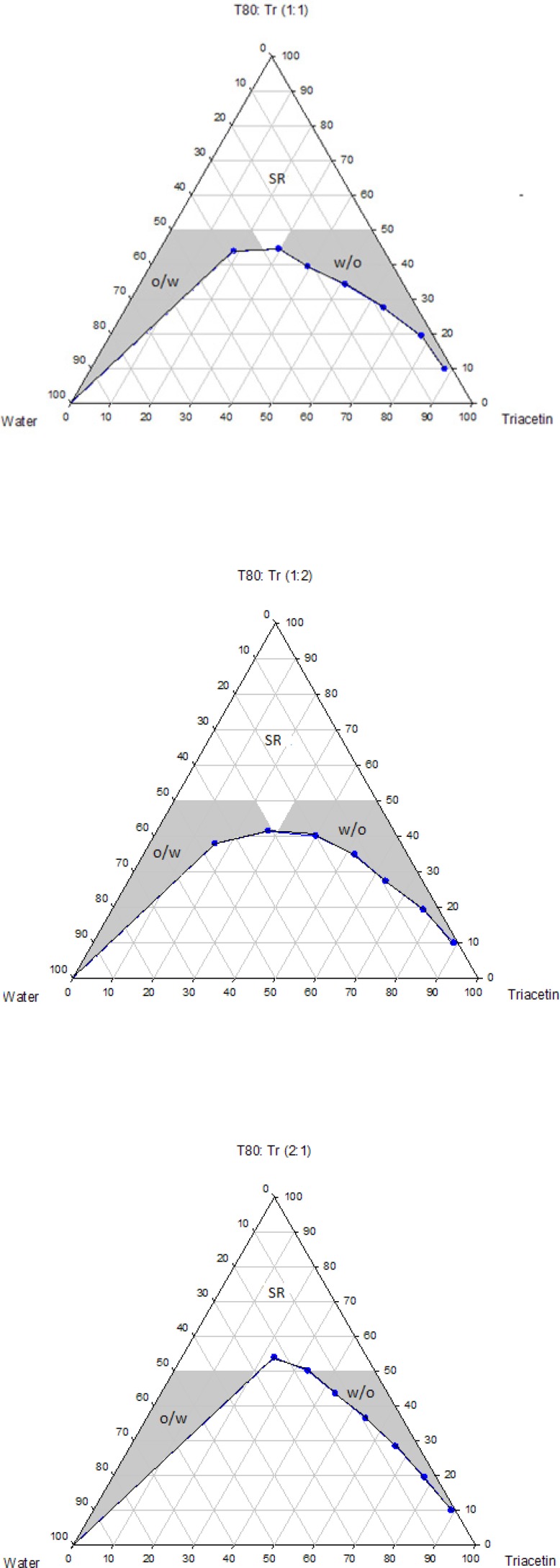
Phase diagrams of the quaternary systems containing Triacetin/Tween 80/Transcutol/water at various R_sm_ (o/w, w/o and SR represent oil-in-water, water-in-oil and surfactant-rich domains, respectively).

**Figure 2:**
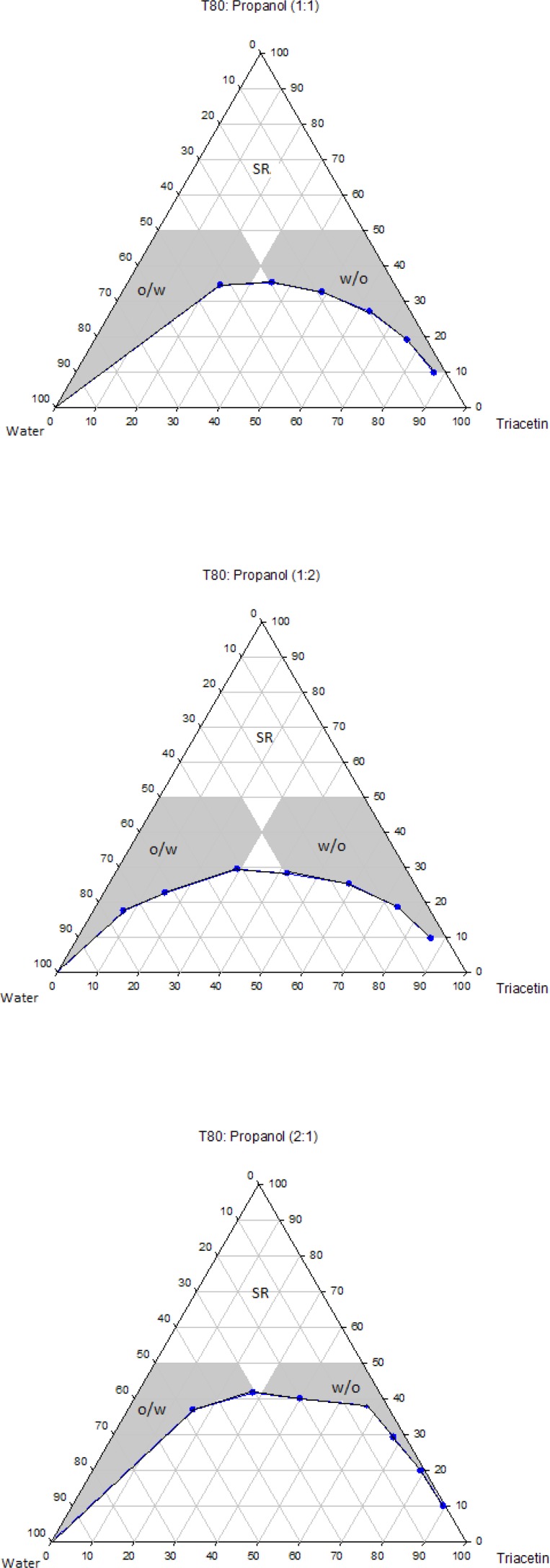
Phase diagrams of the quaternary systems containing Triacetin/Tween 80/*iso*-propanol/water at various R_sm_ (o/w, w/o and SR represent oil-in-water, water-in-oil and surfactant-rich domains, respectively).

**Figure 3:**
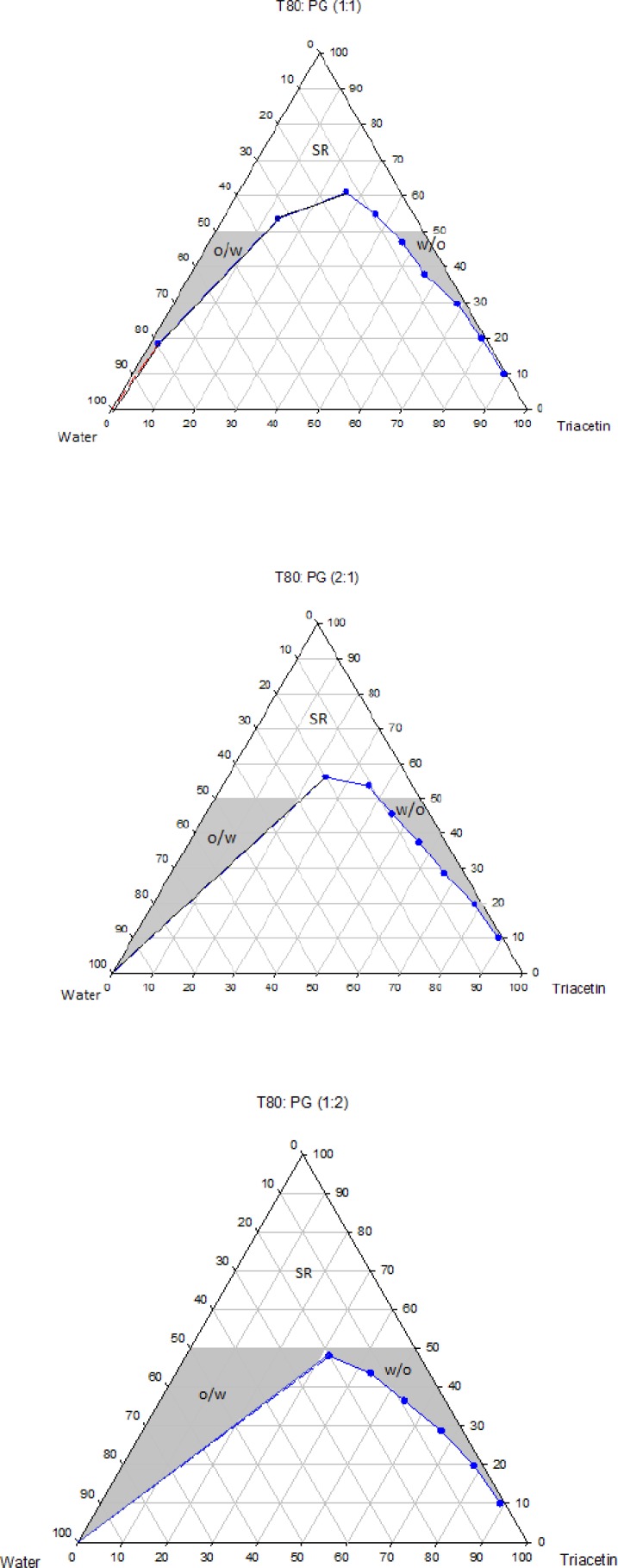
Phase diagrams of the quaternary systems containing Triacetin/Tween 80/PG/water at various R_sm_ (o/w, w/o and SR represent oil-in-water, water-in-oil and surfactant-rich domains, respectively).

**Figure 4:**
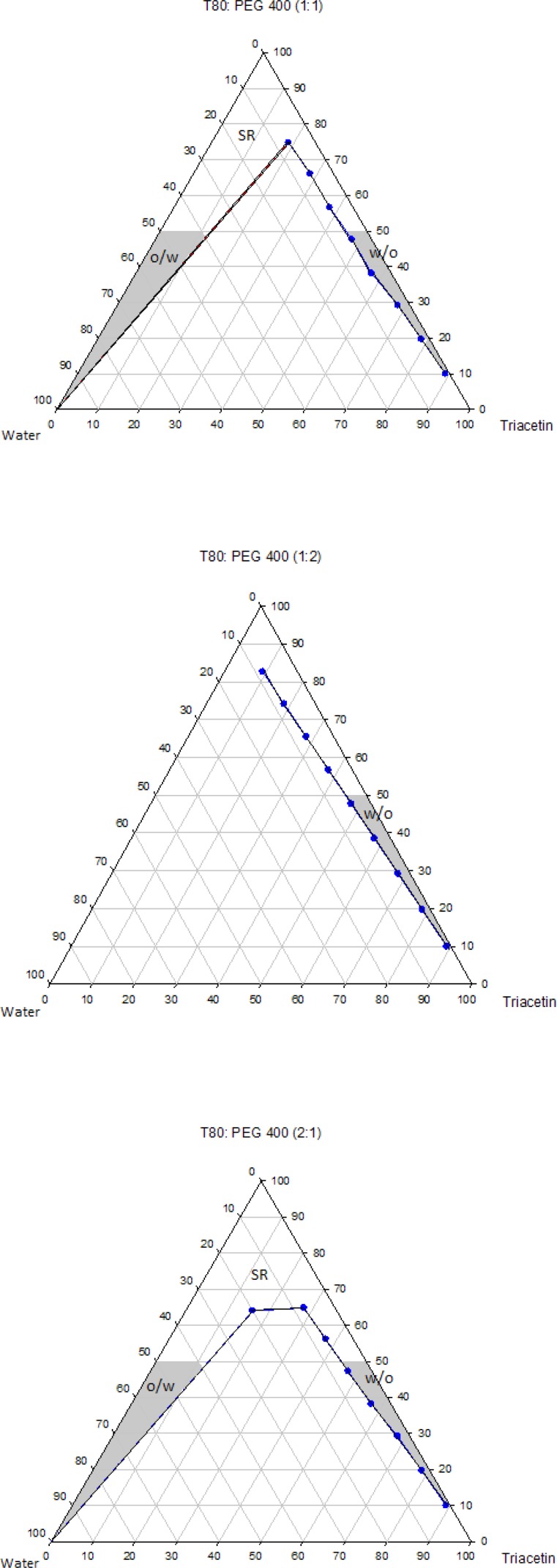
Phase diagrams of the quaternary systems containing Triacetin/Tween 80/PEG 400/water at various R_sm_ (o/w, w/o and SR represent oil-in-water, water-in-oil and surfactant-rich domains, respectively).

The pseudo-ternary phase diagrams of systems containing Triacetin with four different surfactants and co-surfactants at various R_sm_ were constructed. It should be noted that, due to the similarities between the phase behaviors observed with the surfactants studied in order to avoid overcrowding of this paper, only the phase diagrams for Triacetin/Tween 80 are presented. Where appropriate, any differences between phase behaviors observed with other surfactants are mentioned in the text. Figures 1-4 show the diagrams of Triacetin/Tween 80 mixtures in the presence of all co-surfactants. As can be seen, in all phase diagrams (except in Triacetin/Tween 80/PEG 400/water at R_sm_ of 1:2), three different clear, isotropic regions, namely a transparent/translucent domain in the water-rich region, a transparent domain in the oil-rich part and a surfactant–rich (SR) area, designated as o/w, w/o and SRA, respectively, can be labeled, the extent of which depended upon the nature of co-surfactant and R_sm_. It should be noted that because of the difficulties in accurately determining the boundaries between the NE domains and surfactant-rich area, regions with the extension up to 50% w/w surfactant mixture were considered as NEs, above which the area was labeled as SR part.

Although there were some differences between the phase diagrams obtained in this study with various surfactants and co-surfactants, it is beneficial to mention the similarities. In general, the following generalizations can be made about the systems examined:

a) In all systems examined, except in Triacetin/Tween 80/PEG 400/water at R_sm_ of 1:2 and Triacetin/Labrasol/PEG 400/water systems, irrespective of R_sm_, both low viscous o/w and w/o domains were observed.

b) In the presence of any given surfactant and co-surfactant, the change in R_sm_ did not have a significant influence on the extent of NE regions.

c) Regardless of the type of co-surfactant, the change in the nature of surfactant showed a non-significant effect on the extent of NE areas.

d) Regardless of the type of co-surfactant, the extent of o/w domain in the presence of co-surfactants followed the order of iso-propanol > Transcutol > PG > PEG 400.

e) In all systems investigated, no liquid crystal area was observed.

f) Water and oil solubilizing capacities increased with increasing surfactant/co-surfactant content, irrespective of the R_sm_.

g) None of the systems studied, were capable of solubilizing water with less than 10 wt% total surfactant concentrations.

Physicochemical properties of NEs are governed by their compositions and therefore the components must be precisely selected in order to achieve a delivery carrier with desired characteristics (41). In addition to pharmaceutical acceptability which is the most important criterion for the selection of the components, the solubility of the drug in the oil phase is also of considerable importance, since it is greatly influenced by the solubilizing capacity of the oil (42). In this investigation, the solubility of RAP with a Lop P value around 4.3 was determined in Triacetin and found to be 10.86 ± 0.71 mg/mL, while in water it was 2.6 μg/mL. Thus, Triacetin was selected as the oil phase for the development of NE formulations. Another important component of NE systems is the surfactant that stabilizes the interfacial area between oil and water and therefore has a remarkable impact on NE stability. These molecules must be adsorbed rapidly at the at water/oil interface and reduce the interfacial tension to a very small value required for the formation of NE droplets and provide a flexible film around the droplets with an appropriate curvature at the interface (43-45). In addition to these characteristics, it is also important to determine the proper surfactant concentration and use as low concentration as possible. It has been reported that nonionic surfactants are relatively less toxic compared to the ionic surfactants and therefore are preferred for drug delivery (42, 43). As mentioned earlier, in this research, selection of the formulations was based on the criterion of using a minimum concentration of surfactant mixture (*i.e*., 40 wt%). Co-surfactants are also used along with surfactants for the formation of NEs. It has been reported that the addition of these molecules may allow greater penetration of oil in the hydrophobic region of surfactant molecules, further reduce the interfacial tension, increase the flexibility of the interface to take up different curvatures required to form NEs (42, 46-50). Short chain alcohols (*iso*-propanol and PG), Transcutol and PEG 400 are pharmaceutically acceptable ingredients and are commonly added as co-surfactants. Surfactants and co-surfactants are blended in various weight ratios (R_sm_), since it is a key factor influencing the extent and position of NE regions on the phase diagrams. R_sm_ of 1:1, 2:1 and 1:2 were chosen to evaluate the effect of decreasing concentration of surfactant with respect to co-surfactant and the effect of decreasing concentration of co-surfactant with respect to surfactant.

*Selection of o/w formulations from phase diagrams*

As mentioned earlier, those systems indicated a relatively extended o/w NE area on the phase diagrams were selected for the formulation development. It should be noted that based on the extent of NE domain, hundreds of NEs could be prepared from the NE region of the phase diagram. However, it is very important to determine the appropriate surfactant concentration (surfactant plus co-surfactant) and use minimum possible concentration, allowing the access to the oil content in which the drug could be incorporated. In this study, among 48 systems whose phase diagrams were constructed, 23 of them showed the desired feature. Table 1 indicates the components of the selected blank systems. Those formulations that passed the determined criteria in this study (particle size of less than 100 nm, PDI of less than 0.5 and clarity after 72 h storage) were selected for the drug loading and further investigations. Table 1 also depicts the composition of the NEs containing 40 wt% total surfactant and 1 mg of RAP.

**Table 1:** Components of blank nanoemulsion systems selected from phase diagrams and composition of drug-loaded nanoemulsions, at fixed total surfactant concentration of 40 wt%.

**System**	**Components**	R_sm_	**Oil (wt%)**	**Water (wt%)**	**Result** *	**RAP-loaded Formulation**
**S1**	**TAC + T20 + ** ***iso*** **- Prop**	**1:1**	8.98	**51.02**	√	**F1**
**S2**	TAC + T20 + *iso* - Prop	**1:2**	**10.00**	50.00	√	F2
**S5**	TAC + T20 + TR	**1:2**	**8.16**	**51.80**	√	**F5**
**S6**	**TAC + T20 + TR**	2:1	4.30	30.54	√	F6
**S7**	**TAC + T20 + PG**	**1:1**	7.80	50.27	√	F7
**S8**	**TAC + T20 + PG**	2:1	11.30	42.87	√	F8
**S13**	TAC + T80 + *iso*- Prop		2.40	12.45		F13
**S14**	TAC + T80 + *iso*- Prop	**1:1**	4.60	87.12	√	F14
**S15**	TAC + T80 + *iso*- Prop	**1:2**	9.60	45.17		F15
**S16**	TAC + T20 + TR	2:1	2.50	47.01	√	F16
**S17**	TAC + T20 + TR		4.35	59.84	√	F17
**S18**	TAC + T20 + TR	2:1	6.25	74.69	-	F18
**S20**	**TAC + T20 + PG**	**1:1**	11.80	26.14	-	F20
**S21**	**TAC + T20 + PG**	**1:2**	2.60	75.12	-	F21
**S25**	**TAC + Crem RH40 + ** ***iso *** **- Prop**	**1:1**	9.70	48.26	-	F25
**S26**	**TAC + Crem RH40 + ** ***iso *** **- Prop**	**1:2**	14.6	24.25	√	F26
**S29**	**TAC + Crem RH40 + ** ***iso *** **- Prop**	**1:1**	7.60	24.26	-	F29
**S30**	**TAC + Crem RH40 + TR**	**1:2**	2.30	47.65	-	F30
**S37**	**TAC + Crem RH40 + TR**	2:1	10.7	20.64	√	F37
**S38**	**TAC + Crem RH40 + TR**	**1:2**	9.65	50.35	√	F38
**S39**	**TAC + Lab+ iso - Prop**	2:1	**11.80**	48.92	√	F39

Abbreviations: TAC: Triacetin, T20: Tween 20, T80: Tween 80, Lab: Labrasol, Crem RH40: Cremophor RH40, *iso*- Prop: *iso*- propanol, TR: Transcutol, PG: Propylene glycol.

* Systems selected for drug loading.

*Solubility*

Although pharmaceutically acceptable components should be selected for the development of NE-based delivery systems, the solubility of the drug in oil phase of an o/w NE is also an important criterion for the preparation of an efficient NE formulation, because the maintenance of the drug in solubilized form in NE is significantly influenced by the solubility of drug in the oil phase (27, 42). As a consequence of low drug solubility, drug association to the oily phase decreases which in turn, necessitates a higher incorporation of hydrophilic and lipophilic emulsifiers (51). The solubility of RAP in Triacetin was found to be 10.86 ± 0.71 mg/mL, which in comparison to its water solubility (2.6 μg/mL), it represents the potential of this oil to solubilize RAP.

*Particle size and polydispersity index*

**Figure 5:**
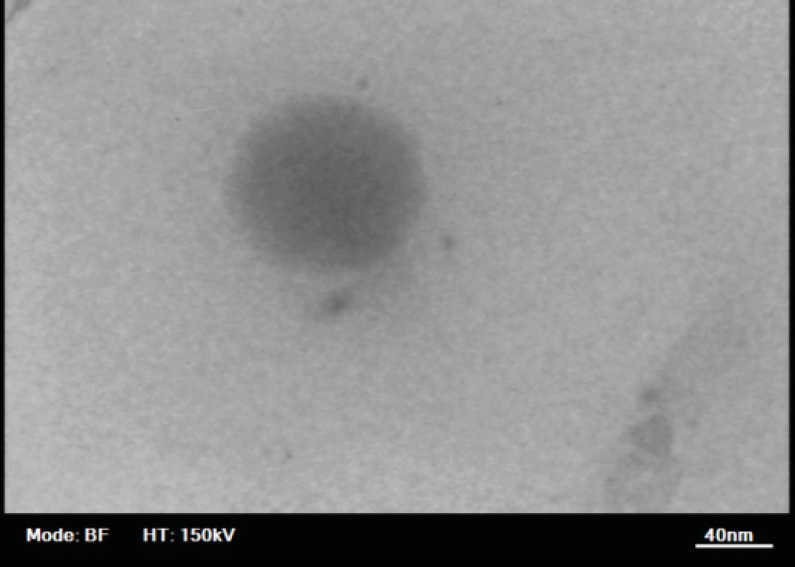
Transmission electron microscopy image of a rapamycin containing nanoemulsion

The droplet size in NEs must be in a nanometer range. In this study, the particle size of the selected drug-loaded NEs was measured using photon correlation spectroscopy to confirm the formation of nanoparticles. In addition, PDI was also determined to provide information about the deviation from the average size. Table 2 depicts the droplet size and PDI values of the selected formulations. It was observed that in all cases, the average size of the emulsions was less than 50 nm. As indicated in Table 2, formulation F6 containing Tween 20/Transcutol at the R_sm_ of 2:1 yielded a nanoparticle diameter of 13.68 nm with a PDI of 0.434, whereas F39 containing Labrasol/*iso*-propanol at the R_sm_ of 2:1 showed a nanoparticle diameter of 47.17 with a PDI of 0.152 which suggests the uniformity of droplet size (47.17 nm) in the formulation. The result of transmission electron microscopy can be observed in Figure 5 which reveals that the lipid emulsion droplets are almost spherical and that the droplet is in the nanometer range.

**Table 2:** Droplet size and PDI value of the selected RAP-loaded nanoemulsions (n=3).

**PDI**	**Z-Average (nm)**	**Formulation**
0.391	13.83	F1
0.455	18.03	F2
0.375	16.51	F5
0.434	13.68	F6
0.416	16.34	F7
0.283	17.83	F8
0.424	20.74	F14
0.380	20.15	F16
0.233	21.64	F17
0.385	26.80	F26
0.442	31.16	F29
0.295	23.98	F37
0.472	31.17	F38
0.152	47.17	F39

*In-vitro release studies*

**Figure 6:**
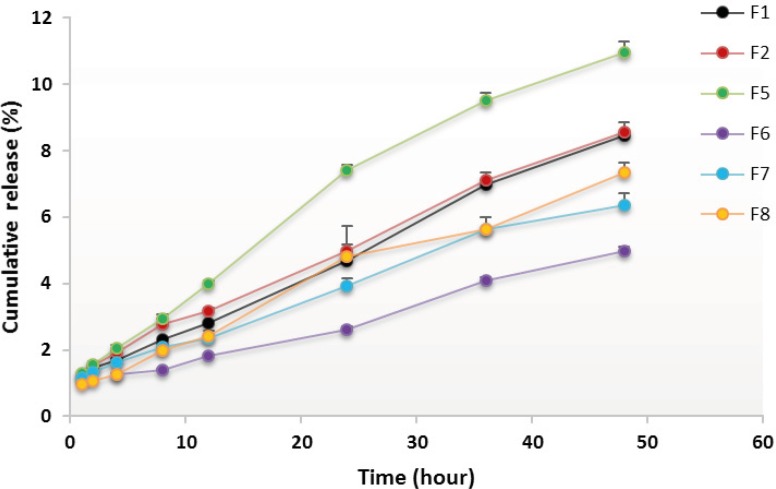
*In-vitro *release profiles of RAP-loaded nanoemulsions composed of Triacetin and Tween 20, from dialysis bag in water containing 0.05 w/v Tween 80 at 37 °C (n=3).

**Figure 7:**
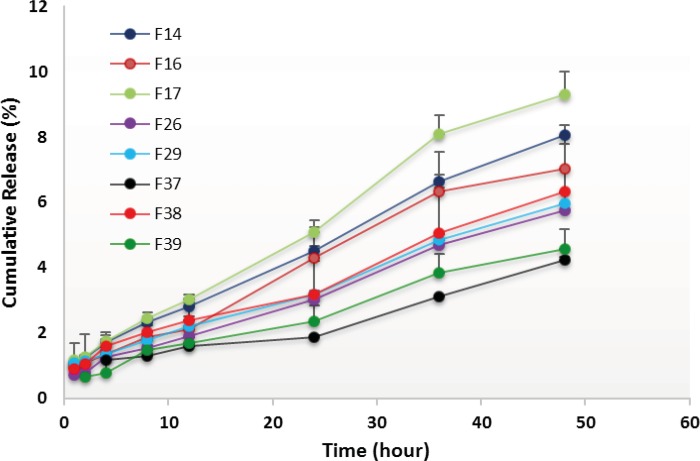
*In-vitro *release profiles of RAP-loaded nanoemulsions composed of Triacetin and three differen surfactants (namely Tween 80, Labrasol and Cremophor RH40), from dialysis bag in water containing 0.05 w/v Tween 80 at 37 °C (n=3).

The use of NEs as lipid-based delivery systems has become progressively popular since most of the new drug molecules are highly hydrophobic (30, 52-54). One of the desired characteristics of a drug delivery vehicle is to provide a sustained release pattern of the solubilized drug. In this investigation, the potential of NE as a drug carrier for RAP was evaluated to ensure whether the drug could be released from formulations in an adequate manner. *In-vitro *release profile of RAP-loaded NEs was therefore studied in water containing 0.05% w/v of Tween 80 at 37 °C and the terminal time point of RAP-release test was selected as 48 h. The mean cumulative percent of RAP released versus time plots are shown in Figures 6 and 7, and the corresponding data obtained after 48 h are presented in Table 3. As illustrated from the plots, in general, the profiles exhibited an initial lag time during the first few hours, followed by a relatively constant slow RAP release within 48 h, showing a typical sustained and prolonged drug release. None of the formulations exhibited complete drug release after 48 h, however, the release rate was found to change depending upon the ingredients added to NEs. 

**Table 3:** Mean cumulative percent of RAP released from nanoemulsions after 48 h (Mean ± SD; n = 3).

**Cumulative release (%)**	**Formulation**
8.5 ± 0.51	F1
8.5 ± 0.33	F2
11.0 ± 0.32	F5
5.0 ± 0.14	F6
6.4 ± 0.38	F7
7.3 ± 0.31	F8
8.0 ± 0.29	F14
7.0 ± 0.99	F16
9.3 ± 0.72	F17
5.8 ± 0.57	F26
6.0 ± 0.35	F29
4.2 ± 0.10	F37
6.3 ± 1.47	F38
4.6 ± 0.62	F39

Plots in Figure 6 show that the highest release, *i.e*., 11 ± 0.32% and the lowest release *i.e*., 5 ± 0.14%, were obtained in cases of F5 and F6, prepared with Triacetin/Tween 20/ Transcutol at the R_sm_ of 1:1 and 2:1, respectively. Statistical analyses indicated that the release of drug from all NE formulations after 24 and 48 h was significant (*p *< 0.05) when compared to the marketed product, Rapamune^®^, which were found to be 1.2 and 2.1%, respectively. On the other hand, comparing the mean cumulative percent of RAP released after 48 h revealed a statistically significant difference between all formulations, except F1, F2 and F8. Plots in Figure 7 depict the release profiles from NEs in which Tween 20 was replaced by other surfactants investigated. The highest rate was obtained in case of F17 (Triacetin/Tween 80/Transcutol at the R_sm_ of 1:2) whereas the lowest rate was observed for F37 (Triacetin/ Labrasol/*iso*-propanol at the R_sm_ of 1:1). Except for F37, a significant increase in the percentage drug release from all NEs was achieved after 48 h as compared to the marketed formulation (*p *< 0.05). Finally, the evaluation of the amount of RAP release from NEs prepared with Tween 80, Labrasol and Transcutol revealed that three formulations, *i.e*., F14, F16 and F17, are statistically significant from other NEs. 

Dissolution studies were performed to compare the release profile of RAP from 14 different NE formulations. In general, the release of the drug from NEs was highly significant when compared to Rapamune^®^ which may be attributed to the high viscosity of the commercial product. It should be pointed out that the viscosity of all NEs was very low as expected. 

Drug delivery potential of NEs may depend upon several key factors including droplet size and polydispersity, viscosity and drug solubility in oil. The existing nanosized droplets lead to enormous interfacial areas. Thereby, in addition to enhancing the solubility of a poorly soluble drug, small globule size and eventually higher surface area in case of NEs would permit faster rate of drug release. Although small differences were observed, however, all of the formulations investigated, had droplets in the nano range (less than 50 nm). PDI indicates the uniformity of droplet size within the formulation. The higher the polydispersity, the lower the uniformity of the droplet size in the formulation. RAP-loaded NEs selected for the *in-vitro *release study showed PDI values less than 0.5, with the minimum value in case of F39 (0.152). Although the particle size results indicated the formation of very small globules, however, the low amount of drug released after 48 h may be explained, in part, by considering the relatively high polydispersity values determined for the NEs.

Lipophilic drugs, like RAP, are preferably solubilized in the oil phase of o/w NEs, depending upon their oil solubility. To develop an efficient o/w NE formulation for such a poorly soluble drug, drug loading in the system is a very crucial factor. NEs with the capability of maintaining the drug in the solubilized form provide reservoir for the sustained drug release. Therefore, in the case of RAP with high lipophilic character (Log P around 4.3), it would be expected that the use of high oil concentration results in lowering the release of the drug into the medium, as the partitioning of the drug will be more towards the oil. On the other hand, Since RAP has a very low solubility in water; the prolonged drug release observed *in-vitro *could be explained by the fact that its diffusion from the oily core and interface is influenced by the aqueous medium. It is noticeable that the dialysis bag used for *in-vitro *conditions could separate the drug containing NEs from the RAP released to the medium and may be in part responsible for this profile (51). The NE formulations (F2, F5, F14 and F17) which showed the highest release profile of drug based on *in-vitro *studies were taken for further investigations. 

*Zeta potential determination*

Zeta potential is a measure of the magnitude of the electrostatic or charge repulsion/attraction between particles that affects the stability. Surface potential (zeta potential) formed by surfactants can produce repulsive/attractive electrical forces among approaching oil droplets and thus prevents their coalescence (55). The more negative or positive the zeta potential, the greater the net charge of the droplets, and the more stable the dispersion is. The results of zeta potential measurements in F2, F5, F14 and F17 NEs presented in Table 4 show a very low positive charge in the range of 0.02-0.9 mv, which is believed to be due to the application of nonionic surfactants. 

**Table 4:** Zeta potential of selected RAP-loaded NEs, based on the release study (mean ± SD; n = 3).

**Formulation**	**F2**	**F5**	**F14**	**F17**
**Zeta potential (mv)**	0.296 ± 0.02	0.019 ± 0.01	0.532 ± 0.04	0.904 ± 0.07

*Stability tests*

**Figure 8:**
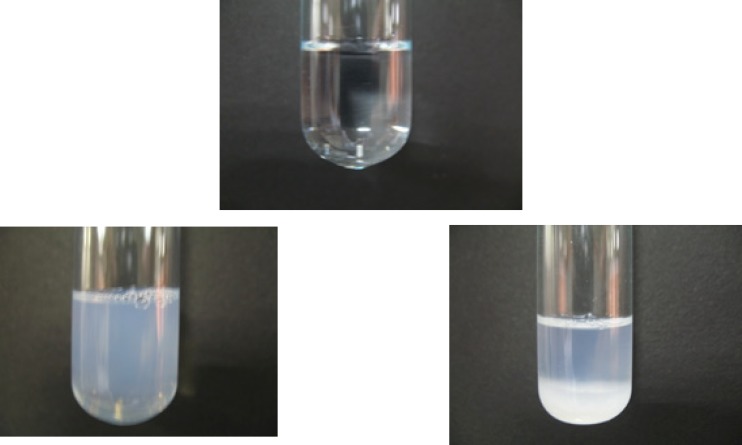
Appearance of nanoemulsion formulations following *a*) 9 months storage at 4 °C, and *b,c*) 12 months storage at 40 °C

To evaluate the NEs stability, the droplet size, PDI and drug content were monitored over 9-12 months storage at 4, 25 and 40 °C. As can be observed in Table 5, at 4 and 25 °C, the NEs presented an increase in mean droplet size (still less than 50 nm) which may be attributed to a good particle stabilization. No phase separation or turbidity was observed. The PDI remained relatively unchanged for all formulations. At 40 °C, none of the formulations survived the stability test and turned to two-phase systems (Figure 8). Stability studies with respect to the assay of RAP in NEs kept at 25 °C (for 12 months) and 4 °C (for 9 months) were analyzed individually (Table 5). The minimum percentage of undecomposed RAP remaining in NE formulations was 21% and 81% at 25 °C and 4 °C, respectively.

**Table 5:** Results of characterization of selected RAP-loading nanoemulsions, following storage at various temperatures for 9-12 months (mean ± SD; n = 3).

**Formulation**	**4 °C**		**25 °C**		**Drug content (%)**	
	**Size (nm)**	**PDI**	**Size (nm)**	**PDI**	**4 °C**	**25 °C**
F2	24.7 ± 1.03	0.423	29.4 ± 2.99	0.465	75.5 ± 3.57	25.2 ± 0.29
F5	37.1 ± 1.04	0.326	31.8 ± 1.99	0.331	81.6 ± 0.84	25.4 ± 0.25
F14	40.2 ± 1.88	0.441	57.5 ± 1.05	0.395	81.4 ± 0.83	26.7 ± 1.2
F17	42.7 ± 0.65	0.266	47.3 ± 0.98	0.301	89.9 ± 0.69	28.0 ± 0.1

* 9-month storage,

** 12-month-storage

*Cytotoxicity assay*

**Figure 9:**
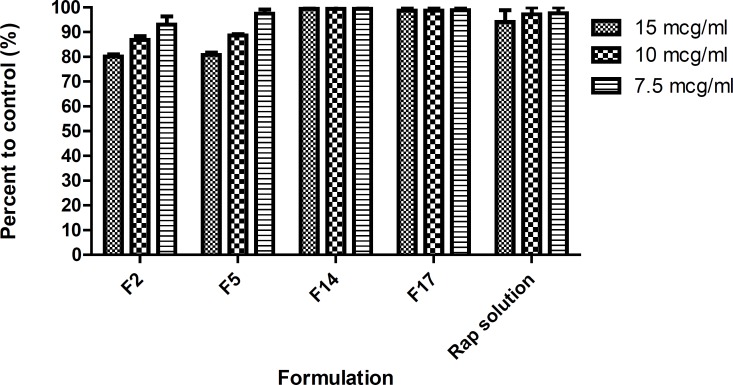
Cytotoxic effect of nanoemulsions containing rapamycin on SKBR- 3 cell line measured by MTT test (48 hour). F2: Triacetin/ Tween 20/*iso*-propanol (R_sm_ of 1:2); F5: Triacetin/Tween 20/Transcutol (R_sm_ of 1:2); F14: Triacetin/Tween 80/*iso*-propanol (R_sm_ of 1:2); F17: Triacetin/Tween 80/ Transcutol (R_sm_ of 1:2) (Mean ± SD; n=3, ** p-value< 0.01, *** p-value < 0.001).

The cytotoxic effect of drug-free NEs was evaluated using MTT test. Results showed that none of the blank NEs were toxic for the SKBR-3 cell line applied in this study (IC_50_> the maximum concentration tried). However, MTT test results revealed that RAP-loaded NEs expressed different toxicity for SKBR-3 cell line, as can be seen in Figure 9. O/w NEs containing Triacetin, Tween 20, *iso*-propanol or transcutol at R_sm_ of 1:2 (*i.e*., F2 and F5, respectively) enhanced RAP delivery to SKBR-3 cell line resulting in more cytotoxic effect (Figure 9). 15-20% of cell death was observed with formulations F2 and F5 which is significantly different from the effect of RAP solution as the control (*p *< 0.001). No change in cell viability was observed when Tween 20 was replaced by Tween 80 (F14 and F17), in comparison with the control, suggesting limited entrance of nanodroplets into the cells. IC_50 _value of RAP methanolic solution was found to be 50 μg/mL and less than 10% of cell death was seen at lower concentrations. Although there is no significant difference between the drug release from F2, F14 and F17 (*p *> 0.05), however, significant difference in cell cytotoxicity was observed (*p *< 0.05). It seems the cytotoxic activity against cancerous cells was not totally due to the direct penetration of free drug into the cells (56). 

15 mcg/mL= 600 μL NE + 400 μL cell culture medium; 10 mcg/mL = 400 μL NE + 600 μL cell culture medium; 7.5 mcg/mL = 750 μL NE +250 μL cell culture medium.

*Transport study*

**Figure 10:**
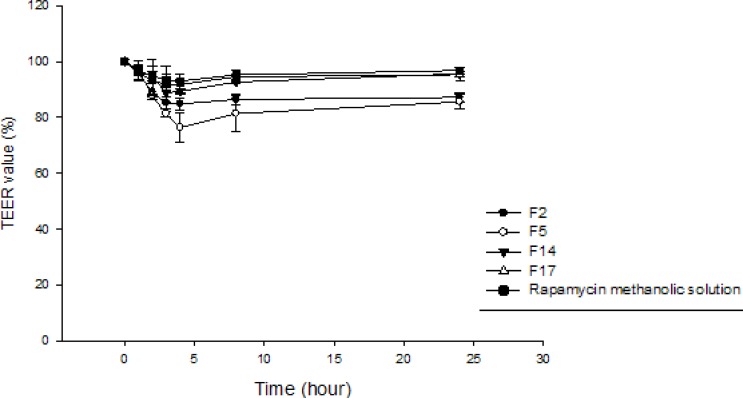
Transepithelial electrical resistance (TEER) values of Caco-2 monolayer measured after the addition of RAP-loaded nanoemulsions and RAP mathanolic solution over a period of 24 hours. (n=3).

**Figure 11:**
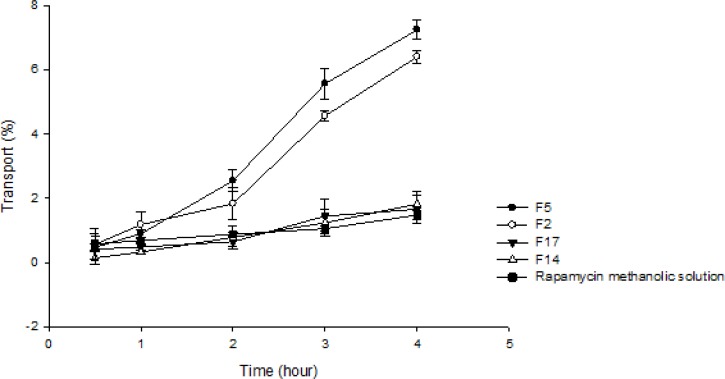
Comparison of apical to basolateral transport of RAP-loaded nanoemulsions and RAP methanolic solution across Caco-2 cell monolayer. The amount of the drug on the basolateral side was measured at the specified time intervals after the addition of drug formulation. The amount of transported drug is expressed as a percentage of the initial drug concentration on the apical side. (n=3).

The development of Caco-2 cell monolayers on polycarbonate filter inserts was investigated by TEER. The TEER value for Caco-2 cells grown on filters after 21 days was calculated to be 600 Ωcm^2^ , indicating the formation of tight junctions and good integrity of the monolayer. TEER was also measured from apical to basolateral side at specific time intervals (1, 2, 3, 4, 8 and 24 h) in the presence of RAP-NEs and RAP methanolic solution. The decline in TEER value after the addition of NEs to the apical side is shown in Figure 10. Formulations containing Tween 20 (F2, F5) caused more considerable decrease in cell integrity in comparison with those prepared with Tween 80 (F14, F17) and methanolic solution suggesting a lower transport of RAP when F14, F17 and methanolic solution were applied (Figure 11). Apparent permeability of each formulation is shown in Table 6. As shown, a significant difference was detected between the transport of RAP-NEs containing Tween 20 and those composed of Tween 80 and methanolic solution. Considering the small particle size and low zeta potential of all formulations, the more apical to basolateral transport may be attributed to the permeability enhancing effect of surfactants used in the structure of NEs. 

**Table 6:** Apparent permeability coefficients of rapamycin-loaded nanoemulsions and methanolic solution of rapamycin

**Formulation **	**P**_app_ **(×10**^-6^ **cm/sec) ± SD **
F2	3.93 ±0.13
F5	4.45 ±0.18
F14	1.02 ± 0.27
F17	1.12 ± 0.23
Rapamycin methanolic solution	0.90 ± 0.09

Paracellular permeability has been examined with Lucifer yellow assays combined with TEER measurements in an attempt to evaluate the effects of polysorbates on human Caco-2 cell monolayers. It has been found that in the paracellular transport experiments, polysorbates altered TEER values and were able to increase Lucifer yellow permeability significantly below the IC_50_ concentration, among which polysorbate 20 has pronounced effect on tight junctions of Caco-2 monolayer (57). The variation of tight junction by polysorbates has also been investigated in human nasal epithelial cell monolayer and the results have shown that polysorbate 80 had no changing effect on tight junction integrity and therefore it was not considered as a good enhancer for paracellular permeability (58).

*Cellular uptake of nanoemulsions*

**Figure 12:**
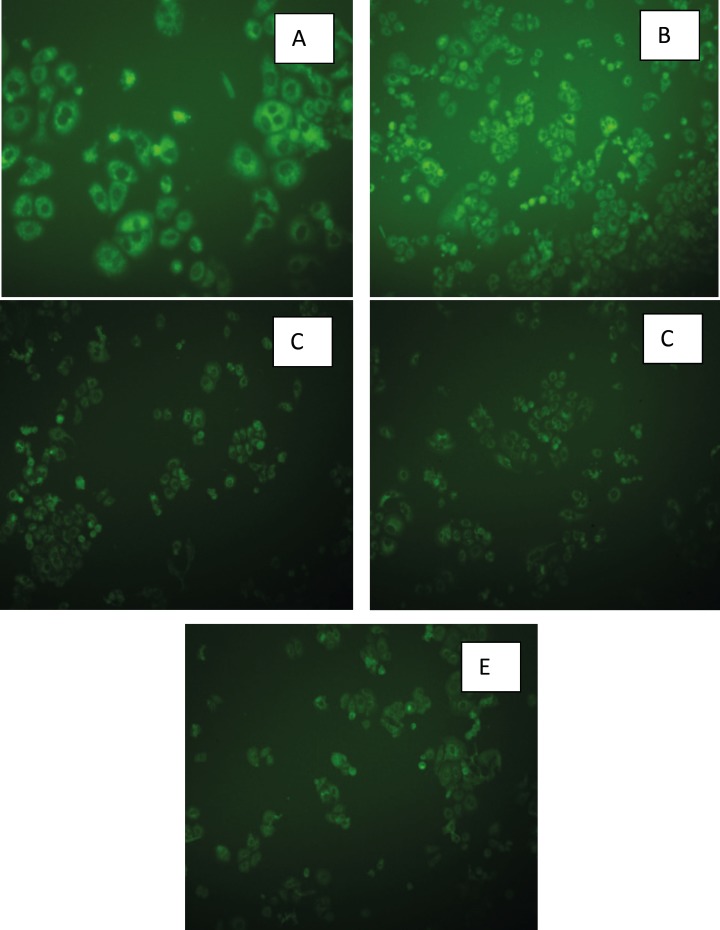
Fluorescent images of SKBR-3 cells incubated for 6 h with A) F2: Triacetin/Tween 20/*iso*-propanol (R_sm_ of 1:2), B) F5: Triacetin/Tween 20/Transcutol (R_sm_ of 1:2), C) F14: Triacetin/Tween 80/*iso*-propanol (R_sm_ of 1:2), D) F17: Triacetin/Tween 80/ Transcutol (R_sm_ of 1:2), E) methanolic solution of coumarin 6.

The results of fluorescent microscopy indicated the cytoplasmic green fluorescence which was considered as the cellular uptake of coumarin 6-loaded NEs and coumarin 6 methanolic solutions by SKBR-3 cells (Figure 12). Fluorescence intensity in different cytoplasmic regions detected from methanolic solution, F14, F17, F2 and F5 was 29.8, 35.53, 31.49, 53.51, 50.62, respectively, which confirmed less cellular uptake in F14, F17 and methanolic solution in comparison with F2 and F5. These results are in agreement with those obtained from TEER and cytotoxicity experiments. 

## Conclusion

Due to the increased interest in cancer therapy, NEs may prove to be promising carriers for drug delivery. Proper selection of components is critical for the development of an efficient nanoemulsion, particularly for drugs with poor water solubility. Oil-in-water Triacetin-mediated NEs were successfully prepared and used for the incorporation of rapamycin (an immunosuppressive agent with anti-proliferative effect) into the oil phase. The cytotoxicity against cancer cells and the enhancement of permeability and concomitant decrease in TEER across Caco- 2 cell monolayer with designed NEs further encourage the use of the these nanocarriers in cancer treatment. However, more investigations may be needed to further understand the efficacy of NEs in drug transport across cancer cells. 
